# Enhanced Mechanical and Corrosion Properties via Annealing Treatment on the Hot-Rolled Ti-Zr-Mo Alloy

**DOI:** 10.3390/ma16072597

**Published:** 2023-03-24

**Authors:** Yun Yue, Mingxing Qi, Tianshuo Song, Bohan Chen, Yihao Tang, Chaoqun Xia

**Affiliations:** 1 National United Engineering Laboratory for Advanced Bearing Tribology, Henan University of Science and Technology, Luoyang 471003, China; 2 State Key Laboratory of Metastable Materials Science and Technology, Yanshan University, Qinhuangdao 066004, China; 3 Tianjin Key Laboratory of Materials Laminating Fabrication and Interface Control Technology, School of Materials Science and Engineering, Hebei University of Technology, Tianjin 300130, China; 4 Department of Mechanical Engineering, The Hong Kong Polytechnic University, Hong Kong, China; 5 State Key Laboratory of Roll Composite Materials, Sinosteel Xingtai Machinery & Mill Roll Co., Ltd., Xingtai 054025, China

**Keywords:** Ti-Zr alloys, microstructure, tensile performance, corrosion resistance

## Abstract

In this work, the Ti-20Zr-15Mo alloy in its hot-rolled state was annealed in different phase zones, and the effects of the annealing treatment on the phase composition, organization, mechanical and corrosion resistance properties of the alloy were systematically investigated. The results showed that the original β grains of the alloy had all recrystallized to form the β equiaxial grains when annealed at 800 °C, and the grains had been significantly refined. This allowed the alloy to reach a tensile strength of 1000 MPa, a maximum of 28% after stretching, and a significant increase in plasticity. Also, due to the single beta phase, there was no galvanic corrosion, making the alloy annealed at 800 °C have the best corrosion resistance.

## 1. Introduction

Titanium and its alloys have the advantages of high specific strength, low density, corrosion resistance, high temperature resistance, and good biocompatibility. Therefore, they play an important role in the aerospace, chemical and medical fields [1,2,3,4,5]. It is well known that Zr and Ti are adjacent elements of the same subgroup with similar physical and chemical properties, and Zr and Ti can be infinitely miscible to produce solid solution strengthening [6,7]. The addition of Zr can improve the plasticity and strength of the titanium alloy while also improving its machinability. In recent years, the development and application of Ti-Zr base has broadened the scope of application of titanium alloys. However, Ti-Zr-based alloys in the as-cast state usually have poor mechanical properties at room temperature, which largely limits their use in important structural materials [8]. In response to this phenomenon, many researchers use thermomachining (such as hot forging, hot rolling, hot extrusion or deformation) to improve strength and meet more engineering applications. Shuang Hu et al. [9] investigated the effects of rolling deformation on the microstructure properties of the Ti-3.5Al-5Mo-4V titanium alloy. The study showed that the larger the rolling deformation, the finer the grain, the deformation increases from 10% to 70%, the strength increases, the plasticity decreases, and at 90%, the strength and plasticity reache the ideal state.

However, the hot-processed specimen has serious defects such as grain deformation orientation, internal stress and dislocation, which cause great difficulties to the subsequent cutting processing of the alloy and hinder its practical application [10,11]. In practical applications, heat treatment deeply affects the organization and mechanical properties of metals. Compared with some new technologies, heat treatment has a low processing cost, high efficiency, and better cost-effectiveness making it more suitable for industrial production. Z.N. Yang et al. [12] mentioned that the β grain size of Zr-2.5Nb is related to the annealing temperature. The higher the annealing temperature, the larger the grain size of the β, because the temperature provides a greater driving force for β phase growth. The higher the temperature, the faster the grain boundary migration rate [13]. Larger grain sizes are often detrimental to the mechanical properties of alloys. So, it is particularly necessary to choose a suitable heat treatment process to regulate the organization and adapt to its performance. S.G. Liu et al. [14] studied the effect of the annealing process on the microstructure and mechanical properties of the TiZrAlB alloy, and the results showed that the microstructure type and grain size determined the mechanical properties of the alloy. Biphasic tissues exhibit the highest intensity, and fine spherical tissue exhibits the best combination of strength and ductility. The various tissues in titanium alloys (equiaxial, reticulated basket, bimorph and Weiss organization) each have their own characteristics and are closely related to the properties of the material [15,16,17]. For example, the plasticity of the two-state organization is generally better; its fatigue strength and creep resistance are better than those of the equiaxial; the net basket organization has excellent high-temperature performance and good creep resistance; and the plasticity of the Weiss organization is poor, generally to avoid its formation. Therefore, it is necessary to study the characteristics and formation mechanisms of microstructure. On the other hand, titanium alloys tend to have insufficient corrosion resistance in harsh environments, and the corrosion resistance mechanism of heat-treated titanium alloys in harsh environments is relatively lacking. Therefore, it is necessary to conduct research in this area.

In previous work, it was found that Ti-20Zr-15Mo exhibited the best mechanical properties when rolled at 800 °C. In order to obtain better strong plasticity, it was annealed in the α single-phase zone (500 °C), α + β dual-phase zone (600 °C, 700 °C) and β single-phase zone (800 °C). The holding time is preset to 10 h in order to give the alloy enough time for recrystallization. The effects of annealing at different temperatures on the organization evolution, mechanical properties and corrosion resistance of the alloy were studied. This provides a theoretical basis for the development of new zirconium-titanium-based alloys and the broadening of their application areas.

## 2. Experimental

Ti-20Zr-15Mo ingots were prepared in a vacuum induction melting furnace (ZKXF-0.005). The Ti and Zr used in the melting were 99.5% pure raw materials, and the Mo used in the alloy was 99.9% pure high purity Mo particles. The specimens were cut into 20 × 20 × 40 mm using EDM wire. The rolled samples with a thickness of 6 mm were obtained by rolling at 800 °C for 30 min and taking multiple passes with a total rolling deformation of 70%. In the previous experiments, the α-phase transition point of the Ti-20Zr15Mo alloy was 535 °C, the biphasic region was from 535 °C to 710 °C, and the β-monophasic region was above 710 °C. The alloy was annealed at 500 °C, 600 °C, 700 °C, and 800 °C for 10 h at different phase zone temperatures.

Samples were sanded with 150#, 600#, 1000#, 2000#, 3000#, 5000# sandpaper and subsequently polished with 1.0 polishing paste. The polished alloys were metallurgically etched using Kroll reagent (H_2_O, HNO_3_ and HF (16:3:1)), and the tissue was observed by (Zeiss Axiovert 200 MAT, Zeiss, Jena, Germany) optical microscopy and (JSM-7100, JEOL, Akishima, Japan) scanning electron microscopy. The elemental distribution was observed with an electron microprobe (EPMA, JXA-8530F, JEOL, Akishima, Japan). The structures of the samples annealed at 800 °C for 10 h were characterized by transmission electron microscopy (TEM, JEOL-2010, JEOL, Akishima, Japan). The phase composition of the samples was analyzed by a X-ray diffractometer (Bruker D8 Discover, Bruker, Bremen, Germany) with a scanning range of 10–90° at 6°/min. Tensile tests were done with sheet specimens in the rolling direction at a stretching rate of 5 × 10^−4^ s^−1^. Tensile fracture morphology was observed by SEM.

The corrosion resistance of the annealed samples was determined in a 5% HCl solution using an electrochemical workstation. The working electrode was the specimen, the silver chloride electrode was the reference electrode, and the platinum electrode was the auxiliary electrode. The specimen exposure area was 1 cm^2^. The experimental open-circuit potential time was 2400 s, and a stable open-circuit potential was observed. The electrochemical impedance spectra at 10^−2^–10^−5^ frequencies was measured under a perturbation signal of 10 mV. Finally, the potential polarization curves were tested at a scan rate of 1 mv/s for the voltage range of −1.5 V–2.0 V.

To ensure the accuracy of the data, three replicate samples were used for all experiments.

## 3. Results and Discussion

### 3.1. Phases and Microstructures

**Figure 1:**
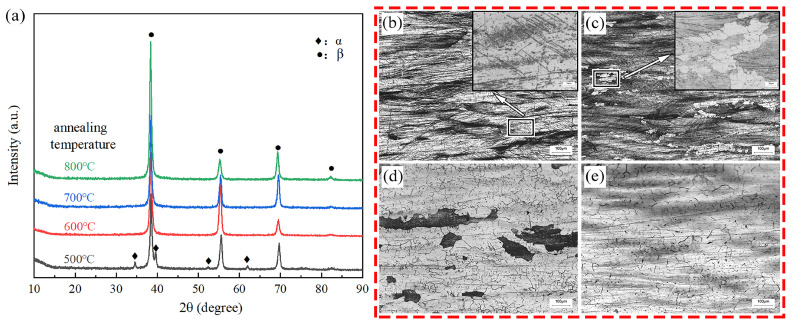
XRD diagram and OM diagram after annealing: (**a**) XRD diagram; (**b**) annealed at 500 °C; (**c**) annealed at 600 °C; (**d**) annealed at 700 °C; (**e**) annealed at 800 °C.

Figure 1a shows the XRD of the alloy after annealing. It can be found that the diffraction peaks of the β phase with BCC structure exist in the alloy, which is due to the high content of the β stable element Mo in the alloy. Therefore, even in the temperature range of the α single phase region, annealing treatment can not completely transform the phase structure of the alloy into the α phase, but there is still some β phase retained at room temperature under the action of the Mo element. This point can be observed in the diffraction peak of the alloy annealed at 500 °C, there are two diffraction peaks of the α and β phases in the spectrum, and the alloy presents a dual phase structure. This phenomenon often occurs when there are strong β-stable elements in titanium alloys. When the alloy is annealed above the α phase transition point, the diffraction peaks show that the alloy phase is all β phase, and no other metal compounds or phase peaks exist. The annealing temperature affects the microstructure of titanium alloys; there are different microstructures at different annealing temperatures, and thus the character shows differences.

Figure 1b–e shows the metallographic microstructure of the alloy annealed at different temperatures. On the whole, the rolled morphology of the sample alloy annealed at 500 °C can be seen, but the difference is that at that temperature it is in the α single-phase region of the alloy. Therefore, the α phase will be preferentially nucleated at the grain boundary during annealing at this temperature, and it can be observed that the alloy forms an acicular α-phase near the grain boundaries due to annealing.

The microstructure annealed at 600 °C shows the formation of recrystallized β-phase small grains near the grain boundaries of the larger grains after rolling. This is because the alloy has a large internal stress after rolling and the grain boundaries can provide the nucleation points and nucleation driving forces of recrystallized β grains, resulting in the morphology shown in Figure 1c. There are secondary β grains near the larger primary β grains. At the same time, it is shown that the morphology of the alloy annealed at 700 °C and 800 °C is basically the same. The matrix is all composed of β grains formed by recrystallization. Therefore, from the results of XRD and metallographic structure, 15 Mo alloy can be fully recrystallized after annealing at more than 600 °C. The long-term heat preservation in the low temperature region can not completely eliminate the morphology of the alloy after rolling, but can only eliminate the deformation stress and rolling defects in the original structure to a certain extent.

**Figure 2:**
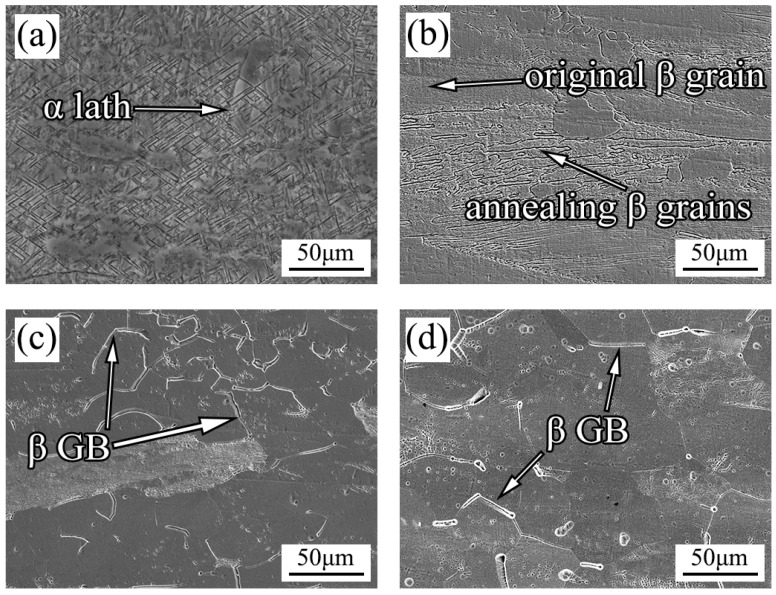
SEM images of annealed Ti-20Zr-15Mo alloys: (**a**) 500 °C; (**b**) 600 °C; (**c**) 700 °C; (**d**) 800 °C.

Figure 2 is the result of further SEM characterization of Ti-20Zr-15Mo annealed alloy specimens. It is shown in Figure 2a that the α-phase laths nucleate and grow from the grain boundary, which is consistent with the XRD results. Since there are many broken grain boundaries after rolling of the alloy, which provides more nucleation points for the α-phase, the annealed laths are smaller than those in the as-cast laths. Meanwhile, due to the low annealing temperature of the alloy, its overall micromorphology is still distributed along the rolling direction. As the annealing temperature rises, the α phase in the alloy disappears, and the whole alloy presents β phase. The smaller β grains of recovery recrystallization precipitate and grow along the grain boundary, while the equiaxed grains distribute along the grain boundary. However, due to the low annealing temperature, the number of equiaxed grains is not much, and there are still large grains along the rolling direction after rolling. The alloy exhibits equiaxed grain morphology at 700 °C. Due to recovery recrystallization, the grain size is obviously refined. Meanwhile, there are only a few large initial grains in the alloy. Compared with 700 °C annealing, the overall morphology of the alloy annealed at 800 °C is similar to that annealed at 700 °C. The equiaxed grains formed by recovery recrystallization are uniformly distributed in the alloy, and the grain orientation morphology after rolling disappears.

**Figure 3:**
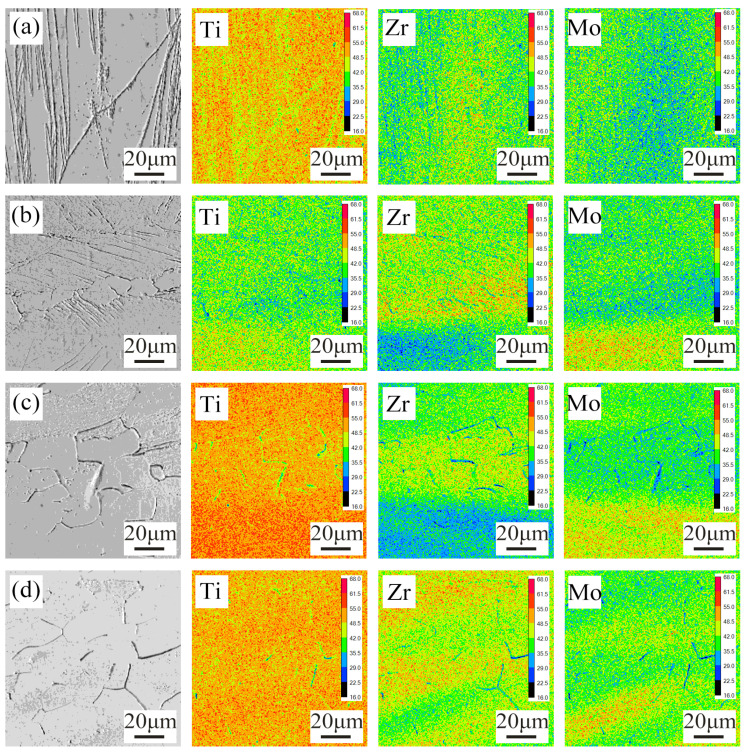
EPMA images of Ti-20Zr-15Mo alloys annealed at different temperatures: (**a**) 500 °C; (**b**) 600 °C; (**c**) 700 °C; (**d**) 800 °C.

The titanium alloy often has a second phase or compound formation after annealing. In order to verify the uniformity of element distribution after annealing, EPMA was used to characterize the alloy. Figure 3 is the characterization result.

EPMA results show that under different annealing temperatures, the alloy has a slight loss of Ti at the grain boundary, but there is no obvious aggregation in the matrix. At the same time, no obvious aggregation or segregation of elements is found in the images of Zr and Mo elements in the matrix, and there is also a phenomenon of low contrast.

Firstly, this phenomenon indicates that there is no intermetallic compound or second phase precipitation at the grain boundary and that the annealing treatment only changes the original phase structure. Secondly, it indicates that the solubility of elements in the alloy at the grain boundary and within the grain is slightly different. The solubility of zirconium and molybdenum elements in the alloy grain matrix is higher than that in the grain boundary.

### 3.2. Mechanical Properties

**Figure 4:**
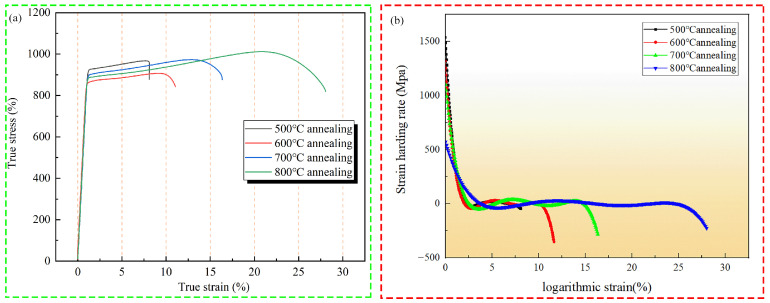
Stress-strain curve diagram and strain hardening diagram of Ti-20Zr-15Mo alloy after annealing: (**a**) true stress-strain curves; (**b**) strain hardening diagram.

**Figure 5:**
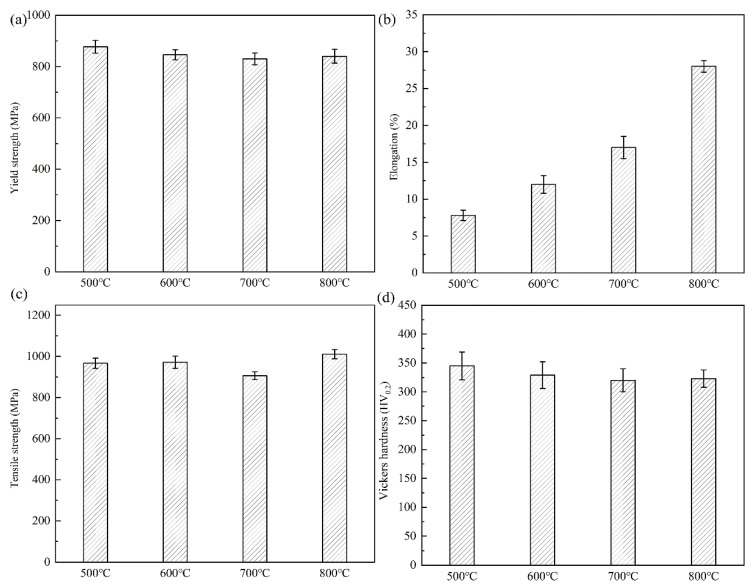
Tensile mechanical properties and Vickers hardness of Ti-20Zr-15Mo alloy after annealing: (**a**) yield strength; (**b**) elongation after fracture; (**c**) tensile strength; (**d**) Vickers hardness.

Figure 4a and Table 1 shows the true stress-strain curves of the alloys after different annealing treatments. As can be seen from the curve, the yield strength of the annealed alloy decreases, and elongation is positively correlated with annealing temperature. After annealing at 500 °C, the alloy has a yield strength of 880 MPa, but it shows a low plasticity level of about 6%. This is because the microstructure of the alloy after annealing at this temperature is the dual-phase structure of the rolled β grain and the α phase lath distributed along the grain boundary. Moreover, the β phase slip system of the BCC structure is greater than that of the α phase slip system of the HCP [18,19,20]. Dislocations will pile up near the grain boundary and the slip system will decrease, resulting in local stress concentration. With the continuous accumulation of tensile dislocations, the alloy generates microcracks in this region and expands to the interior of the grain. Thus, the alloy after annealing at 500 °C exhibits a higher yield strength and poorer plasticity. As the annealing temperature rises, the yield strength of the alloy decreases. After annealing at 600 °C, the yield strength is about 840 MPa, and the elongation after fracture reaches 18%. This is because the microstructure of the alloy at this temperature is all β phase. The phase of the alloy is uniform, and the alloy has a BCC structure with many slip systems. Dislocation density decreases with increasing annealing temperature. However, due to the lack of phase, which has a certain obstacle effect on dislocation except at grain boundaries, the yield strength of the alloy is reduced. The yield strength of the alloy annealed at 700 °C is further reduced to about 820 MPa, but the plasticity is reduced by 4% compared with that of the alloy annealed at 600 °C. The reason for this decline in plasticity may be that the annealing temperature of the alloy cannot reach complete recrystallization, and there are still large original β grains and small grains formed by annealing in the sample. It can be seen from the metallography that the sizes of the two kinds of grains are quite different, so the deformation incoordination occurs in the tensile deformation course, resulting in the early fracture of the alloy [21]. The yield strength of alloy specimens after annealing at 800 °C did not decrease significantly; it was still about 820 MPa, but the plasticity increased by about 28%. This is because the alloy undergoes a phase transition and the organization changes to a single β-phase structure. The BCC structure with its more slip system is prone to yielding and plastic deformation under stress. At the same time, the grains formed under complete recovery and recrystallization at high temperature were refined. According to the Hell-Petch formula, small grain size would reduce the effective slip length of dislocation, which was more beneficial to the plasticity of the alloy [22]. Figure 4b shows the strain hardening rate graph. All alloys show a similar trend of variation. The hardening rate decreases rapidly at the beginning of deformation, which corresponds to the elastic phase of the stretching process. In the middle of the deformation, as the deformation process will produce dislocation plugging, this will lead to a certain rise in the hardening rate; and the deformation process will generate heat, which will to some extent resist the ability of the alloy plastic to deform, this process is called thermal softening. Thermal softening is generally manifested as a decrease in strain hardening rate, and the mechanism of simultaneous action of thermal softening and process hardening maintains the strain hardening rate at a stable level. In the later stages of deformation, the hardening rate becomes negative when necking occurs, accompanied by the sprouting and expansion of cracks within the alloy, and finally fracture occurs [23,24]. Since the dislocation density decreases after annealing, the strain hardening rate does not rise significantly after the elastic stage, and a stable strain hardening rate curve is formed. Figure 5a–d shows the yield strength, elongation after fracture, tensile strength, and hardness of the annealed alloy. As can be seen from the graphs, the alloys annealed at 800 °C have good elongation and tensile strength. However, the hardness of the alloy after annealing at 500 °C is higher than that of other annealing temperatures. This is because the alloy still has a large rolling deformation stress at this temperature, which provides a certain contribution to the hardness. Annealing also affects the dislocation density, and at high temperatures the dislocation density is low, which also makes the hardness decrease. As the annealing temperature increases, the hardness of the alloy is reduced to varying degrees, indicating that the internal stress of the alloy is gradually eliminated.

**Figure 6:**
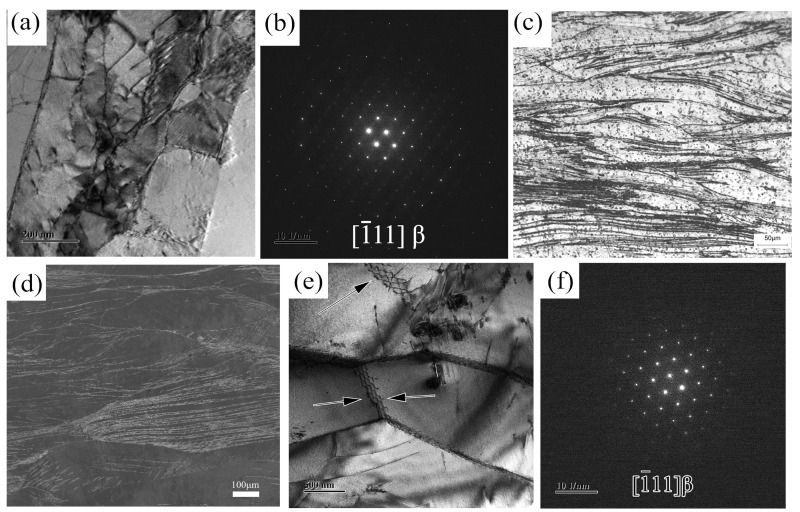
(**a**) rolling alloy: TEM image; (**b**) rolling alloy: diffraction spots; (**c**) rolling alloy: OM image; (**d**) rolling alloy: SEM image; (**e**) 800 °C annealing alloy after the tensile test: TEM image; (**f**) 800 °C annealing alloy after the tensile test: diffraction spots.

**Figure 7:**
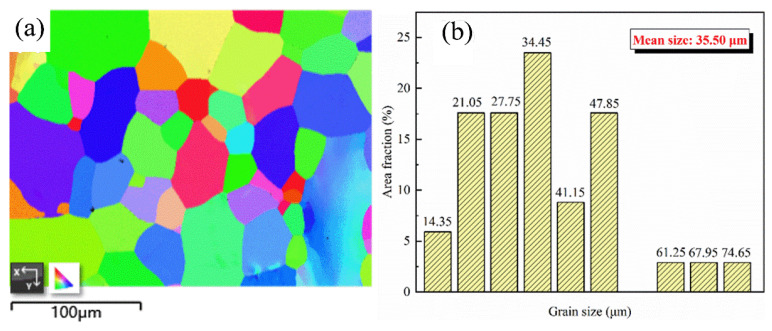
800 °C annealing alloy: (**a**) EBSD image; (**b**) grain size statistics.

The TEM images obtained after rolling and annealing after the tensile test are shown in Figure 6. Figure 6a–d shows the correlation diagram of the alloy after rolling. The rolled alloy has more dislocations and intertwined grain boundaries. This is because the dislocations are moved downward by compressive stress during the rolling process and gather at the grain boundaries, forming a region of high stress and laminar dislocation energy. Figure 6e shows the TEM image of the alloy after annealing at 800 °C after a tensile test. More residual dislocation lines and dislocation bands formed by the stacking of dislocation lines were found in the region pointed by the arrow. The image was observed when the tensile strain of the alloy reached about 14%. It is shown in the figure that dislocations form dislocation stacking near the grain boundaries, and small angle grain boundaries are formed by a large number of dislocation stacking. It can be proved that the grain boundaries of the alloy after annealing at 800 °C have a strong ability to retain the dislocation lines, and the grain boundaries formed by dislocation stacking can increase the activation energy of the secondary start of the dislocation so that the alloy has better mechanical properties. The diffraction spots in Figure 6f show that the alloy is in the β phase of the BCC structure. Figure 7a presents the EBSD image of the alloy after annealing at 800 °C. It is clear that the average size of equiaxed β grains formed by recrystallization of the alloy after annealing in the β single-phase region is 35.50 μm (Figure 7b). Therefore, the recrystallized grains formed by annealing at 800 °C are equiaxed grains, and the grain size is relatively uniform and significantly refined compared with the original β grains as cast. Therefore, the annealed alloy at this temperature can have better plasticity and yield strength.

**Figure 8:**
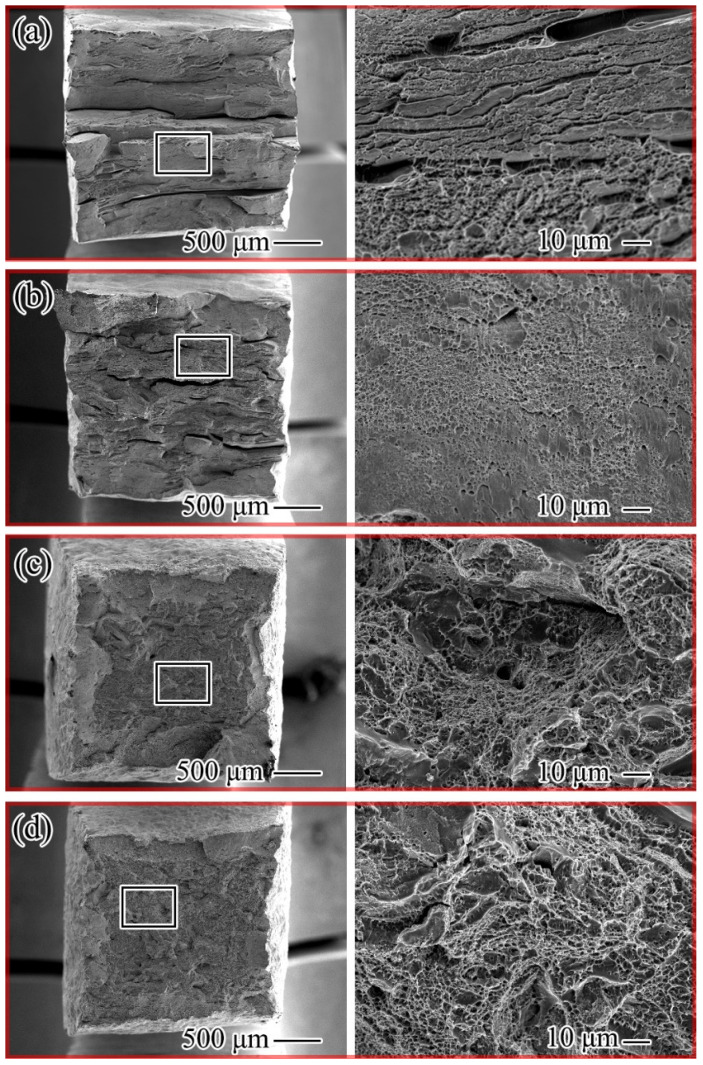
Fracture morphology of the annealed Ti-20Zr-15Mo alloy: (**a**) 500 °C; (**b**) 600 °C; (**c**) 700 °C; (**d**) 800 °C.

Figure 8 shows the macroscopic and microscopic images of the tensile fracture morphology of the alloy after different annealing processes, and the right side is the left side of the local amplification image. The fracture microstructure of the alloy sample annealed at 500 °C shows uneven distribution, and there are large cracks. The local detail amplification diagram demonstrates the existence of complete grain boundaries, and the overall characteristics are consistent with intergranular fracture, which is one of the reasons for the poor plasticity of the alloy sample.

Figure 8b is the fracture morphology annealed at 600 °C. The existence of dimples can be observed in the detail amplification diagram. At the same time, it can be seen that the cleavage plane or cleavage step is relatively smooth in the fracture. There are many cleavage planes in the fracture, which is characterized by a brittle cleavage fracture. The fracture morphology that annealed at 700 °C is displayed in Figure 8c. From the macroscopic image of the left side, it is evident that the tensile fracture of the alloy is not the crack shown by the previous two annealing processes. The existence of dimples can be seen in the enlarged image, and the cleavage surface is smaller, which is a feature of ductile fracture [25,26,27]. In the fracture morphology after annealing at 800 °C (Figure 8d), no cracks are evident in the macrostructure of the alloy, which is the same as that exhibited in Figure 8c. However, the figure shows that the alloys dimple distribution is uniform and the overall surface is smooth. This is due to the thorough annealing of the alloy at this temperature without the original coarse β grains. After complete annealing, the β grain size is uniform, so it has better coordination deformation ability and presents more excellent plasticity.

### 3.3. Corrosion Behavior

**Figure 9:**
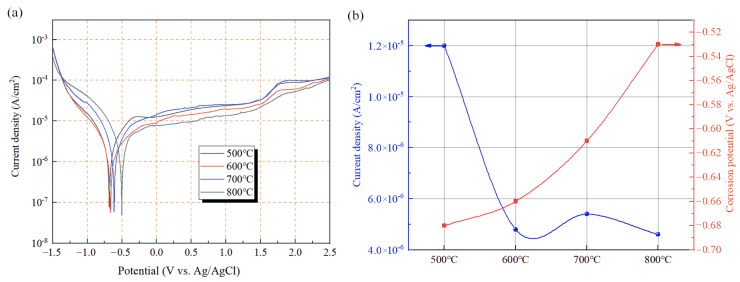
(**a**) Polarization curves of Ti-20Zr-15Mo alloys at different annealing temperatures; (**b**) trend of corrosion potential and corrosion current density.

The tafel polarization curves of Ti-20Zr-15Mo alloy after annealing at 500 °C, 600 °C, 700 °C and 800 °C are shown in Figure 9. From the initial −1.5 V scan to the self-corrosion potential is the cathodic region of the reaction. It can be seen from the figure that the shape of the cathodic curves of the alloy specimens annealed at different temperatures are similar. This indicates that the annealing treatment has not changed the cathodic reaction tendency of the alloy. After the self-corrosion potential, with the continuous increase of voltage into the anodic region, I_corr_ in a very small voltage range appeared to increase rapidly, indicating that the active polarization occurred on the alloy surface in the electrolyte, but the duration of the region is short and the active polarization is completed quickly [26]. With the increasing scanning potential, the corrosion current density of the alloy remains flat for a period of time. At this time, the alloy surface passivation film is dissolving and generating under the application of external voltage to maintain a dynamic equilibrium. The larger dimensional passivation interval of the alloy can be seen from the figure, which is due to the passivation metal of the titanium alloy. The Zr and Mo elements in the alloy enhance the protection of the passivation film and thus enlarge the dimensional passivation interval. However, after this interval, except for the alloy annealed at 800 °C, all three alloy specimens showed a rapid increase in I_corr_ after the dimensional passivation interval. This phenomenon indicates that the dynamic balance between the generation and dissolution of the alloy’s passivation film is broken, the passivation film is broken, the alloy substrate reacts directly with the solution, the protective effect of the passivation film fails, and the corrosion current density of the alloy increases rapidly. By this time, the surface of the alloy specimen has uniformly developed pitting phenomena. It is important to note that the pitting potential is the stage at which the pits develop uniformly and is not the potential at which pitting begins. The appearance of pitting in an alloy is related to the internal organization of the alloy and is not influenced by the state of the passivation film. Unlike the remaining three alloy specimens, the 800 °C annealed specimens showed a slow increase in I_corr_ and did not show a rapid rise. The lack of rapidly developing pitting suggests that uniform dissolution of the passivation film occurs in this alloy and that this leads to an increase in corrosion current density.

The alloy annealed at 500 °C has the highest I_corr_ and the lowest E_corr_ among the four annealed alloys, and the reason for this phenomenon is related to the physical phase of the alloy. This is due to the alloy being annealed to show α + β two-phase structure in the alloy between the different phases of the potential difference between the microcell, thus causing the galvanic corrosion to intensify the corrosion process. Therefore, the alloy specimens annealed at 500 °C exhibit poorer corrosion resistance. As seen in Figure 9b, the corrosion current density of the other three samples with single β phase is of the same order of magnitude, with little difference between them. In addition, it is noted in the literature that in Titanium alloys, β is more likely to form passivation films and protect better than α slats [27]. Secondly, the organization and morphology of the alloy have different effects on the corrosion performance of the alloy. The 800 °C annealed alloy specimens are equiaxed, and the grain distribution is uniform at this temperature and the alloy shows optimal corrosion resistance. Soltis [28] and others have studied the mechanism of passivation film formation in titanium alloys and found that passivation film nucleation in titanium alloys has a selective tendency to nucleate preferentially at defects such as grain boundaries. The smaller the grain size, the easier the alloy passivation film can be generated to protect the substrate. Due to incomplete annealing at 600 °C and 700 °C, there are still large original β grains that have not been fully recrystallized. Therefore, an alloy annealed at this temperature has lower corrosion resistance than an allot annealed at 800 °C.

When a zirconium alloy is immersed in a solution containing chloride ions, a corrosion crater is created, and the corrosion dissolution in the crater can be expressed by the following reaction equation:

(1) $$M+{\mathrm{aCl}}^{-}+{\mathrm{bH}}_{2}O\to M{\left(\mathrm{OH}\right)}_{b}{\mathrm{Cl}}_{a}^{\left(-b-a+4\right)+}+{\mathrm{bH}}^{+}+4e^{-}$$

where M denotes the metal element and *a* and *b* denote the constants. When immersed in the Cl^−^ solution, local acidification will occur within and/or around the pits. As a result, Cl^−^ will migrate to the vicinity of the pit to maintain the electrical neutrality between the corrosion pit and the solution, thereby increasing the corrosion rate of the alloy. From the polarization curves, the pitting of the alloy annealed at 800 °C is less pronounced than that of the alloy annealed at other temperatures. It indicates that the alloy annealed at 800 °C suppresses the pitting phenomenon to some extent.

In addition, the polarization behavior was further analyzed using the polarization resistance *R_p_*. Its value is calculated from the tafel diagram using the Stern-Geary formula:

(2) $$R_{p}=\frac{babc}{2.303I_{\mathrm{corr}}\left(ba+bc\right)}$$

where *ba* is the anodic tafel slope and *bc* is the cathodic tafel slope, and the polarization resistance indicates the resistance of the material to general corrosion. The corresponding parameters are shown in Table 2.

**Figure 10:**
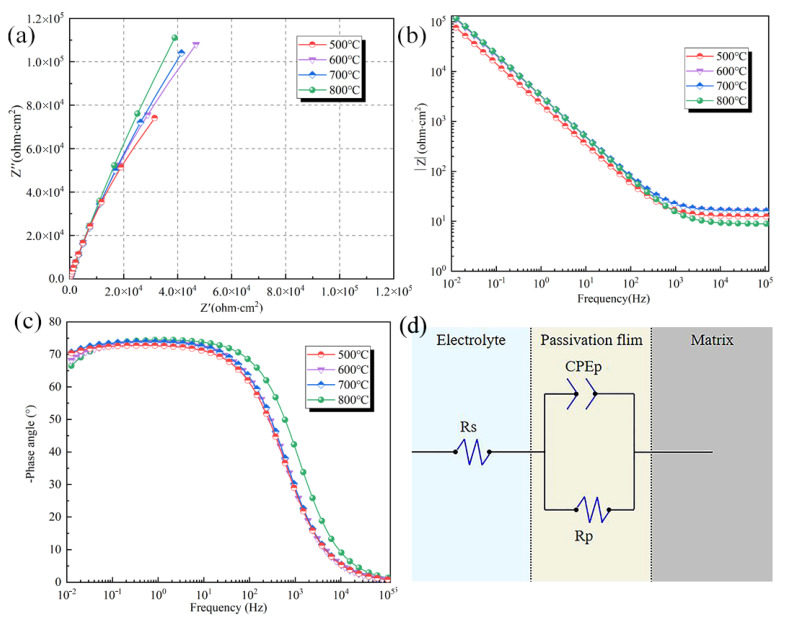
Electrochemical impedance spectroscopy of Ti-20Zr-15Mo alloys after annealing: (**a**) Nyquist plots; (**b**) Bode impedance plots; (**c**) Bode phase angle plots; (**d**) equivalent circuit diagram.

To evaluate the protective effect of the passivation film on the substrate surface, electrochemical impedance spectroscopy was used for the corresponding study. The electrochemical impedance of Ti-20Zr-15Mo alloy was studied at different annealing temperatures in a 5% hydrochloric acid solution. Nyquist and Bode plots are shown in Figure 10a–c, respectively. The performance of the alloy passivation film can be evaluated by fitting the equivalent circuit model, and the equivalent circuit model shown in Figure 10d was selected for the fitting analysis according to the degree of fit [29,30,31,32]. From Figure 10a, Nyquist diagrams of all alloys consist of a semicircular arc, which is the capacitive arc of impedance, and only one semicircular arc indicates that there is only one time constant controlling the reaction process of the alloy. The surface alloy passivation film has only one layer. At the same time, the radius of the capacitive arc is a good reflection of the resistance value of the alloy passivation film, and the larger the radius of the capacitive arc, the more obvious the protection of the substrate. The variation of the capacitive arc with temperature in the Figure 10. The pattern is consistent with the dynamic potential polarization curve. From Figure 10b, the slope in the high frequency band is 0, which is attributed to the response of the electrolyte solution, which also corresponds to the Rs phase inside the circuit diagram. In the low and mid frequency region, the slope is maintained at −1, which corresponds to the capacitive response of the passivation film. This also corresponds to the middle part of the circuit diagram. The intersection point with the *Y*-axis in the low frequency region represents the impedance value of the film, and it can be seen that the 800 °C annealed impedance value is the largest, indicating a good corrosion resistance. The phase angle of the low and middle frequency region in Figure 10c is maintained, which also corresponds exactly to the low and middle frequencies of the Bode impedance diagram.

Table 3 shows the electrochemical impedance spectrum data. The data show that the solution resistance values all fluctuate to a small extent. It means that there was no more dissolution of corrosion products in the electrolyte during the test, and the solution. There is no large fluctuation in the impedance test process, and the test environment is stable. The data in the table shows that the polarization impedance value is proportional to the annealing temperature, which indicates that the increase in annealing temperature has a positive effect on the corrosion resistance of the Ti-20Zr-15Mo. The data in the table show that the maximum polarization resistance (3.32 MΩ·cm^2^) was obtained for the specimens after annealing at 800 °C. According to Mark Orazem’s article [33], the capacitance value can be calculated using the following formula:

(3) $$\mathrm{Ceff}=Yo^{1/n}R^{\left(1-n\right)/n}$$

The magnitude of the Ceff and *n* values represents the stability of the alloy surface passivation film. When the Ceff value is lower and n tends to be close to 1, the alloy passivation film can effectively block the contact between the solution and the substrate, thus providing stable protection [34,35]. At the same time, the thickness of the oxide film can be easily estimated from the effective capacitance value using the following formula:

(4) $$C=\frac{\varepsilon \varepsilon_{0}S}{d}\;$$

where *ε* is the dielectric constant of the passivation film and *ε*_0_ is the vacuum dielectric constant of 8.8542 × 10^−14^ F/cm; S is the corresponding area of 1 cm^2^. Since it is a sample with more titanium content, the dielectric constant of the passivation film is chosen as the dielectric constant of TiO_2_. The relevant calculation parameters are shown in Table 3. According to the trend of the n value and the polarization resistance value in Table 3, the passivation film structure on the alloy surface is effectively improved with increasing annealing temperature. At the same time, the high annealing temperature results in fewer defects in the passivated film. Among the specimens annealed at several annealing temperatures, the alloy specimen obtained by annealing at 800 °C has an n value of 0.92 and an effective capacitance value of 5.16 × 10−5 F-cm^2^. In addition, the estimated oxide film thickness is the largest. Both of which are the best among the four annealed specimens and thus have the best corrosion resistance performance, which corresponds exactly to the results of the kinetic potential polarization curve. The error values of the fits are all below 2.0 × 10^−3^, with high confidence. From the impedance data, the alloy annealed at 500 °C has the smallest resistance, and the polarization curve also has the largest self-corrosion current density and the smallest polarization resistance at 6527 Ω. These two sets of data coincide, indicating that the alloy annealed at this temperature has the worst corrosion resistance. However, the resistance value calculated from the polarization curve at 800 °C is also the highest, at 29,843 Ω. This also corresponds to the impedance spectrum.

## 4. Conclusions

In this paper, the effects of different annealing temperatures on the organization, mechanical properties and corrosion resistance of hot-rolled Ti-20Zr-15Mo alloy were systematically examined. The following conclusions were obtained:

1. The grain orientation characteristics left by the rolling treatment are not eliminated after annealing at 500 °C, and there is pin-like α-phase precipitation along the initial β grain boundary. The grain orientation of the alloy is gradually eliminated with increasing temperature. When the annealing temperature is 800 °C, all the original β grains of the alloy have been recrystallized to form β equiaxed crystals, and the grains are obviously refined.
2. With increasing annealing temperatures, the plasticity of the alloy increases but the strength decreases. The tensile strength after annealing at 800 °C reaches 1000 MPa, and the elongation after fracture reaches a maximum of 28%. This is because the microstructure formed by annealing at this temperature is a fine, equiaxed grain structure, which produce an obvious fine grain strengthening effect.
3. The corrosion resistance of alloy annealed at 500 °C is poor. This is because annealing at 500 °C causes the formation of galvanic coupling corrosion between the α-phase and β-phase, which accelerates the corrosion of the alloy in hydrochloric acid and thus shows the worst corrosion resistance. The 800 °C annealed alloy with fine β equiaxial crystals exhibited the best corrosion resistance.

## Figures and Tables

**Table 1:** Yield strength, compressive strength and elongation after fracture of the annealed Ti-20Zr-15Mo alloys.

Alloys	500 °C	600 °C	700 °C	800 °C
Yield strength (MPa)	880 ± (16)	840 ± (17)	821 ± (6)	820 ± (9)
Ultimate tensile strength (MPa)	980 ± (12)	986 ± (13)	900 ± (9)	1006 ± (8)
Elongation (%)	6 ± (1.7)	14 ± (0.3)	18 ± (0.5)	28 ± (0.4)

**Table 2:** Polarization curve corrosion parameters.

	E_corr_ (V)	I_corr_ (A/cm^2^)	*ba* (mV/Decade)	*bc* (mV/Decade)	*R_p_* (Ω)
500 °C	−0.68	1.2 × 10^−5^	406	341	6527
600 °C	−0.66	4.8 × 10^−6^	674	322	19,711
700 °C	−0.61	5.3 × 10^−6^	378	260	12,620
800 °C	−0.53	2.1 × 10^−6^	326	259	29,843

**Table 3:** Corrosion parameters of the annealed Ti-20Zr-15Mo alloy from EIS data in a 5 wt.% HCl solution.

Alloys	Rs (Ω·cm^2^)	Y0 × 10^−5^ (s^n^/Ω/cm^2^)	Ceff × 10^−5^ (F/cm^2^)	d nm	*N*	Rp (MΩ·cm^2^)	χ2(×10^−3^)
500 °C	11.84 ± 0.02	2.67 ± 0.07	7.47 ± 0.10	0.56	0.81 ± 0.10	3.02 ± 0.11	1.26 ± 0.12
600 °C	8.67 ± 0.07	2.90 ± 0.15	7.28 ± 0.13	0.58	0.83 ± 0.08	3.09 ± 0.12	1.54 ± 0.09
700 °C	8.78 ± 0.11	3.27 ± 0.12	5.80 ± 0.09	0.73	0.89 ± 0.07	3.18 ± 0.09	0.92 ± 0.14
800 °C	9.86 ± 0.07	3.85 ± 0.15	5.16 ± 0.14	0.82	0.92 ± 0.05	3.32 ± 0.016	1.06 ± 0.21

## Data Availability

The data used to support the findings of this study are included within the article.
